# The Influence of Work Resources, Demands, and Organizational Culture on Job Satisfaction, Organizational Commitment, and Citizenship Behaviors of Spanish Police Officers

**DOI:** 10.3390/ijerph17207607

**Published:** 2020-10-19

**Authors:** Alexandra Marcos, Cristina García-Ael, Gabriela Topa

**Affiliations:** Department of Social and Organizational Psychology, National Distance Education University UNED, 28040 Madrid, Spain; amarcos129@alumno.uned.es (A.M.); cgarciaael@psi.uned.es (C.G.-A.)

**Keywords:** work demands, work resources, organizational culture, job satisfaction, organizational commitment, organizational citizenship behaviors

## Abstract

The present study aims to analyze the influence of work demands and resources (support and control) on the attitudes and behaviors (satisfaction, organizational commitment, and organizational citizenship behaviors toward the organization, OCBO) of Spanish police officers, and to examine the potential mediating role of the flexibility-oriented organizational culture. Participants were 182 Spanish police officers. The analysis was carried out using the Smart PLS (Partial Least Squares) program. Firstly, reliability and convergent and discriminant validity were analyzed. Secondly, the structural model was evaluated. Overall, findings support the hypothesized model, except there was not a significant effect of demands and support on OCBO (Organizational Citizenship Behavior Organization-oriented). Results of the importance-performance map analysis also show that, in terms of predicted job satisfaction and organizational commitment, control and support are not so important, but both of them perform relatively well compared to the remaining constructs (demands and flexibility-oriented culture).

## 1. Introduction

Nowadays, police organizations face a wide range of environmental pressures and police officers should attempt to tailor their operations and services to the community’s needs [1]. In order to perform their functions by maximizing the results of their efforts, police officers’ physical and psychological wellbeing should be considered as a relevant topic [2,3,4], since empirical evidence has shown the link between personal wellbeing and positive outcomes, such as job satisfaction, organizational commitment, and citizenship behaviors [5,6]. Furthermore, any organizational effort to alter the work environment should be also reflected in the culture of the organization and be transmitted through organizational socialization. In this vein, several studies have shown that, when desirable results are achieved, the process of organizational socialization leads to an individual integrated into the working group, able to cope with work demands, who knows which resources are available and where to find them [7]. Furthermore, an accurately socialized person can understand where and how work demands and available resources may vary and to what extent the flexibility of the organizational culture allows every individual to adapt their performance to the specific requirements of each situation. This enables employees to engage in organizational citizenship behaviors, to be committed to their job position and workgroup, and to be satisfied with their work [8]. Theoretical models have indeed integrated the analysis of work demands and resources [9,10], mainly perceived support and control [11,12], together with the socialization process [13].

In the present study, we aim to explore how can this idea of enhancing personal potential in the work environment by using group culture influence among a specific group of people: police officers. The choice has been made to highlight not only the paramount importance of this public institution for every modern society but also because of its characteristics, which can provide innovative information for the current article. At the individual level, police officers have to deal every day with decisions that can lead to citizens’ life or death, freedom or captivity, health or damage; thus, it is understood that the responsibility both towards society and individuals is high, as is stress, given the face-to-face service police officers develop at work. At the organizational level, there is already a “police culture” in the institution, considering, among others, the specific language for tools and procedures, or specific abilities and permissions conceded by the law very different from other occupations with a key role in people’s lives. Most important, however, is the feeling of a “profound corporate unity” with deep group ties based on the belief of belonging to a special collective and a solid unity that will act when problems arise.

In sum, this study aims to analyze the influence of work demands and resources (support and control) on the attitudes and behaviors of Spanish police officers, in particular on their job satisfaction, their organizational commitment, and their organizational citizenship behaviors, as well as the potential mediating role of a flexibility-oriented organizational culture. This model will be tested under the theoretical umbrella of positive organizational psychology and be focused on promoting employees’ wellbeing [14]. Finally, as in previous studies in Spain [15], we attempt to clarify the way by which positive personal results would be improved in both the short and long term.

### 1.1. Work Demands, Support, and Control

The job demand control (DC) model [11] and the subsequent extension, the demand-control-support model [12], have been used on many occasions to assess job characteristics and job stressors [16]. Besides, both have served as a basis for the development of other models or theories, such as the job demands-resources model (JD-R) [17]. The DC model proposes that interactions between the various levels of work demands and job control are the reason for different types of psychological experiences at work and can result in high- and low-strain jobs and active and passive jobs [18] (p. 31).

High-strain jobs are a combination of high work demands and low job control, whereas low-strain jobs are the opposite: low work demands and high job control. Passive jobs are characterized by low work demands and low job control, while jobs characterized by high work demands and high job control are referred to as active jobs. On the other hand, the demand-control-support model adds the workplace social support dimension that buffers the negative effects of job strain [12]. Both approaches have been applied to different variables such as cardiovascular risk [12], disorders of the digestive system [18] (p. 135), and workplace bullying [19].

Other research has also focused on organizational outcomes and behaviors. For example, high-strain jobs have been related to higher levels of workplace harassment [20] and a low level of psychological empowerment and job satisfaction [21]. Likewise, Fink and Schaubroeck found that the interaction between work demands, social support, and job control predict health symptoms, absence of disease, organizational commitment, and satisfaction with job supervisors [22]. In the same vein, Hagedoorn and Van Yperen suggest that control reduces fatigue in jobs with high demands, whereas high levels of social support cause high levels of intrinsic motivation, regardless of demand and control levels [23]. Empirical research showed that strain in a police officer can be referred to as high for all the abovementioned characteristics of everyday work, and so having a social source of support would be quite interesting for the institution, preventing absence and other harmful consequences [24,25,26,27].

### 1.2. Job Satisfaction

Job satisfaction is a positive and global emotional response to one’s work as a whole [28]. Job satisfaction is often synonymous with overall or global satisfaction and is evaluated through items that ask people how much they like their work. In general, the influence of affective dimensions of work on different organizational outcomes, such as the individual performance of employees and the achievement of organizational goals, is recognized [29,30]. As a result, interest in the assessment of job satisfaction is as current as it was when the first affective indicators of job satisfaction were proposed almost 60 years ago [31].

### 1.3. Organizational Commitment

Organizational commitment is a construct that highlights the link between the individual and the organization, involving “the acceptance of goals and values of the organization” [32]. The difference between engagement and commitment should be taken into account: the former refers to a motivational concept, while the latter is rather attitudinal [33].

In this vein, the current study considers affective commitment to the organization as a result of the socialization process, which includes the emotional link that binds the individual with the organization and that leads to feeling identified, like living organizational problems as your own and being proud of the collective success [34]. As has been already mentioned, national police can have some particularities, as it is 24-h work, given the legal requirement for police members of any rank to be available to act anytime, anywhere, solving citizens´ issues or situations included in their legal and social role.

### 1.4. Organizational Citizenship Behaviors

Organizational citizenship behaviors (OCB) are the behavior towards the organization and refer to individual behaviors that are not directly and explicitly recognized in the formal reward system. OCB is not behaviors required in the work contract nor the job role, but are personal choices, and their omission is not punishable. OCB improves organizational effectiveness by helping to create a psychological, social, and organizational context in which employees perform their job responsibilities [35]. It is a fact that today’s high performance and work productivity reign in any organizational context. Nevertheless, it has been proved that OCB is more valued by supervisors than even the target performance within the organization [36]. For this reason, there is no doubt that OCB is highly attractive both from the point of view of management evaluation and in terms of organizational performance.

Here, it is important to analyze how a situation like facing criminals and working together knowing that the lives of the members of the group depend on their performance can influence this variable because an extraordinary trust is necessary and would be beyond the formal role and job description.

### 1.5. Flexibility-Oriented Organizational Culture

Organizational culture is a set of key values and norms shared by members of an organization [37]. Organizational culture has often been linked to job satisfaction, organizational citizenship behaviors, and the retention of qualified employees [38]. For the purpose of this study, organizational culture is considered based on the competing values approach [39], a model encompassing two dimensions with opposite values: (a) internal vs. external orientation and (b) flexibility vs. control. The first dimension is related to organizational focus (internal: micro emphasis on the wellbeing of people in organizations vs. external: macro emphasis on the wellbeing of the organization itself). The second value refers to the level of flexibility—that is to say, the trend of decentralization and differentiation in decision-making (flexibility) or the tendency towards stability.

The combination of the two dimensions generates four organizational culture orientations: support, innovation, rules, and goal orientation. The flexibility dimension is defined by the combination of support and innovation and is characterized by adaptability to change and innovation in task development. As a result, a flexibility-oriented organizational culture includes openness and innovation orientation, support for individual development, and a support orientation characterized by personal confidence [40,41]. The dimension of flexibility is particularly relevant to understanding complex working contexts such as the field of policing. In fact, the daily situations in which police carry out their tasks demand spontaneity, adaptability to change, openness, responsiveness, and innovation to address issues that do not fit a specific description provided in advance. For this reason, it can be expected that a flexibility-oriented organizational culture influences the job satisfaction and performance of Spanish police officers.

## 2. Study Aims

Spanish National Police, founded by King Ferdinand the VII in 1824, is guided by values like discipline and rank order (Organic Law 2/1986, 13 March). Nowadays, Spanish police officers have to swear an oath of being available permanently (24 h a day) to assist citizens. Furthermore, certain positions require working abroad (e.g., embassies) or working away from their units for months due to reasons of national and public security, irrespective of the area they work (Scientific, Information, Immigration and borders, Judiciary and Public safety). Different studies carried out in Spain have shown that police officers are highly likely to suffer from work-related stress due to the tasks of their work [1], such as responding to emergency calls, arresting violators, delivering warrants, mediating conflicts between citizens promptly, which, in turn, harms their physical and psychological wellbeing [2,3,4]. Although previous research has also shown that flexibility-oriented cultures exert their positive effects on employees’ job satisfaction (among others) [15], as far as our knowledge reaches, there is an absence of empirical studies focused on how a flexibility-oriented organizational culture can reduce the negative effects of work demands of Spanish police officers, that is to say, facilitate positive outcomes at the employee level. With this aim in mind, we attempt to contribute to a theoretical extension of the research on the job demand control model [12,17,18,21] through the integration of the flexibility orientation in organizational culture, which has not been sufficiently explored in the past and can serve as a stimulus for future research.

Accordingly, the present study investigated whether the relationship between job demands and resources (support and control) and the attitudes and behaviors of Spanish police officers (job satisfaction, organizational commitment, and OCBO) would be mediated by flexibility-oriented organizational culture. Based on the previous studies carried out with the job demand control (DC) model and the subsequent extensions [23,26,30,31,34], it was hypothesized that work demands will directly predict job satisfaction (H1a), organizational commitment (H1b), and organizational citizenship behaviors (H1c). Second, it was hypothesized that perceived support at work will directly predict job satisfaction (H2a), organizational commitment (H2b), and organizational citizenship behaviors (H2c). Likewise, it was hypothesized that job control will directly predict job satisfaction (H3a), organizational commitment (H3b), and organizational citizenship behaviors (H3c). Additionally, based on previous research [15] it may be expected that a flexibility-oriented organizational culture mediates the relationships between work demands and job satisfaction, organizational commitment and organizational citizenship behaviors (H4a), support and job satisfaction, and organizational commitment and organizational citizenship behaviors (H4b), and between job control and job satisfaction, organizational commitment and organizational citizenship behaviors (H4c).

**Figure 1:**
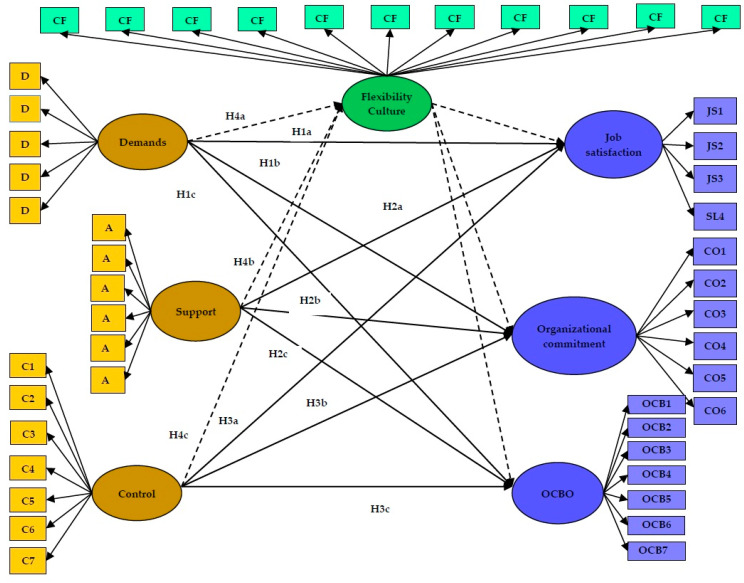
Hypothesized model **Note**: OCBO: Organizational Citizenship Behavior Organization-oriented. The potential mediations of the flexibility-oriented culture are marked with a discontinuous line.

All the hypotheses proposed in this study are shown in Figure 1.

## 3. Method

### 3.1. Participants and Procedure

The sample consists of 182 Spanish police officers (59% men), ranging from 24 to 61 years old (*M* = 43.8; *SD* = 8.7), with an average of 16.9 (*SD* = 9.7) years of service in the organization. Forty percent of them (41.6%) had completed university studies, 27 percent professional or vocational training, and 31 percent high school education_._ Police officers came from three different regions of Spain (Comunidades Autónomas): Madrid (46.15%; *n* = 84), Castilla y León (29.13%; *n* = 53) and Castilla La Mancha (24.72%; *n* = 45). Participants differed in rank: 5 percent belonged to upper management, 30 percent to executive management and the remainder (64%) were police officers. The Police Academy helped to contact three regional police forces. Executive serving police officers of each municipality were informed clearly and accurately about the purpose of the research, the procedure to be used, and the time required to perform the questionnaire. After this, police members were contacted by e-mail (and reminder mailing) to inform them about the study. The questionnaires were administered by the main investigator who went to each police unit during the different shifts to apply the questionnaires, which were completed during working hours. Participants first completed the registration page and the consent form and then filled out the survey on paper. Besides, the main investigator provided a large envelope in which police members could place their questionnaires once completed in order to ensure anonymity and confidentiality. The administration of the questionnaire took about 15 min. The response rate for surveys remained 30 percent (30.33%). Adequacy of the sample is based on the ratios by gender and hierarchical balance in the whole of the organization (mostly composed by men—about 9500, which represents the 15% based on data collected in 2019 by the Internal Affairs Ministry, in which the organization is included—and also more numerous in the police officer position—in about 65.000 police officers in the organization, about 46.000 belong to the basic police officer category, which is also according data collected in 2019 by Internal Affairs Ministry).

### 3.2. Instrument

To measure work demands, perceived social support, and job control, the Spanish validation [42] of the Job Content Questionnaire (JCQ) [4] was used. This questionnaire comprises three dimensions: psychological demands (six items), job control (seven items), and job support (eight items). For each item, the response was recorded on a five-point Likert scale, ranging from 1 (strongly disagree) to 5 (*strongly agree*).

The psychological demands scale refers to workload (e.g., “I am not asked to do an excessive amount of work”), work intensity (e.g., “my job requires working very hard”), time pressure (e.g., “my job requires working very fast”), cognitive complexity (e.g., “My job requires a high level of skill”) and conflicting work (e.g., “I am free of conflicting demands that others make”). The job control scale measures the ability to manage work demands and resources available at work (e.g., “my job allows me to make a lot of decisions on my own”). Finally, the work-related social support scale is the sum of two subscales: support from supervisors (e.g., “my supervisor is concerned about the welfare of those under him”) and support from co-workers (e.g., “people I work with take a personal interest in me”).

Job satisfaction was measured by the Spanish validation [43] of the Brief Index of Affective Job Satisfaction [44]. The original scale comprises seven items (three of them distractors) related to emotional satisfaction as a global and positive emotional response to the position in general. For the study, however, we took only the four relevant items (e.g., “I feel very satisfied with my work”, “I thoroughly enjoy this work”). The alpha value obtained in our study was 0.86, greater than in the original study with distractors (0.83) [43]. Responses are rated on a five-point Likert scale ranging from 1 (strongly disagree) to 5 (strongly agree).

The chosen scale to assess the organizational commitment variable was the Spanish validation [45] of the commitment scale developed by Allen and Meyer [34]. This 24-item scale measures affective, continuance, and normative commitment. For the purposes of the study, however, we selected affective commitment (seven items, e.g., “I would be very happy to spend the rest of my career with this organization”) because it was the most relevant approach to this construct, and the heart of organizational commitment is attributable to the emotional ties that bind the individual to the organization in which they work. Responses are rated on a five-point Likert scale ranging from 1 (strongly disagree) to 5 (strongly agree).

To assess organizational citizenship behavior, we used the Spanish validation [46] of the Organizational Citizenship Behaviour Scale [47] composed of two subscales: behaviors directed towards individuals (OCBI) and toward the organization (OCBO), with eight items each. In the study, we used the OCBO because previous research showed the highest associations with other organizational variables such as job satisfaction [47,48,49]. Using a five-point scale ranging from 1 (never) to 5 (always), participants were asked to indicate how often they engaged in these behaviors (e.g., “Attend functions that are not required but that help the organizational image”).

To test the flexibility-oriented culture, we used the Spanish validation [50] of the FOCUS 93 questionnaire based on the competing values model [39]. Following Azanza et al. [15], the flexibility-oriented culture dimension was measured with six items from the support scale (e.g., “How often do management practices allow freedom in work?”) and five items from the innovation scale (e.g., “How often does your organization search for new markets for existing products?”). A five-point Likert scale from 1 (never/nobody) to 5 (always/everyone) was used.

In terms of socio-demographic data, data related to survey participants’ age, gender, educational level, seniority, and hierarchic level within the organization were collected.

### 3.3. Data Analysis

Descriptive statistics and correlations between the variables of the study were examined. Additionally, data were analyzed using the SEM method using Smart PLS 3.2.6. software focused on predicting dependent variables, latent and manifest; maximizing the explained variance (R^2^) of the dependent variables; and reducing the residual variance of endogenous variables in any regression run of the model [51]. Smart PLS estimates the standardized regression coefficients for the different relationships established in the model between observable indicators and latent variables, as well as between the different latent variables.

Two sorts of results are provided: indicators about the psychometric properties of constructs are obtained (outer model), and relationships between the different latent variables can be simultaneously analyzed (inner model). Following previous research [52], PLS has two strengths that make it well suited to this study. PLS was developed to avoid the need for large sample sizes and hard assumptions of normality [53]. For this reason, it is often referred to as a form of soft modeling [54]. PLS is also recommended for situations where a theory or model is to be built [53]. Significance was assessed using bootstrapping of 500 samples, which led to a critical t-value of 1.96 for *p* < 0.05. Finally, we conducted an importance-performance map analysis (IPMA) for each criterion variable with Smart PLS in order to provide information on the relative importance (and performance) of constructs. The IPMA provides performance scores on a scale from 0 to 100.

## 4. Results

### 4.1. Preliminary Data Analyses

In order to test if there were any relevant differences according to the participants’ gender, age, or organizational tenure, ANOVA’s tests have been conducted following the procedure used by Recio and colleagues [55]. There were no differences according to gender (*p* > 0.05) in any of the variables included in the model (Work Demands, Social Support, Control, Flexibility-Oriented Culture, Job Satisfaction, Organizational Commitment, or OCBO). Concerning the police officers’ age, considering two age groups (above and below 45 years), we could not find significant differences in any of the independent nor dependent variables. Concerning the participants’ organizational tenure, categorized into two groups (above and below 15 years of seniority), we found no differences in the independent or dependent variables. Finally, concerning the status of participants (1 = upper and executive management vs. two police officers), we found no significant differences in the majority of the variables (Work Demands, Social Support, Flexibility-Oriented Culture, Organizational Commitment, Job Satisfaction, OCBO (all *p*_s_ > 0.05), except for the variable control (*M*_1_ = 3.47 *SD*_1_ = 0.63 vs. *M*_2_ = 2.94 *SD*_2_ = 0.82; *p* < 0.05). These results are consistent with previous research showing that job content is different across posts [25]. In other words, police officers encounter significantly more physical psychological threat-related events (e.g., dealing with family disputes or crises) than executive management and, subsequently, they could feel or perceive having less control over their work.

### 4.2. Descriptive Statistics and Pearson’s Correlations

Descriptive statistics (means and standard deviations) and correlations between the variables of the study were examined (Table 1 and Table 2).

The analysis in PLS was carried out in two stages [55]. Firstly, tests of reliability and convergent and discriminant validity were analyzed (outer model); then the hypotheses were tested (inner model)—that is to say, to what extent perceived demands, support, and control shown by Spanish police officers predicted their job satisfaction, organizational commitment, and OCBO. It was also examined whether these relationships were mediated by a flexibility-oriented culture.

### 4.3. Reliability and Validity of the Constructs: The Outer Model

The individual reliability of each indicator was given by the loadings between the indicator and the construct (λ). As shown in Table 3, the standardized outer loadings were higher than 0.60 [43], except for three items (D2, CF3, CF7). Nevertheless, these items were kept because of their theoretical significance. Composite reliability (*ρc*) and Cronbach’s alpha were estimated to evaluate the internal coherency of all the indicators related to the construct: *ρc* is a preferred alternative to Cronbach’s alpha to measure the internal consistency reliability because Cronbach’s alpha assumes that all indicators are equally reliable, whereas PLS prioritizes indicators according to their reliability, resulting in a more reliable composite [40]. The cut-off for acceptable *ρc* and Cronbach’s alpha is 0.70 [56]. Therefore, results indicate that all the constructs exceeded the minimum requirements.

To assess convergent validity (common variance between the indicators and their construct), the average variance extracted (AVE) was used. The higher the AVE value, the more representative the indicators are of the construct on which they load. Generally, the value should be above 0.50 [55,56]. As presented in Table 3, the AVE for each construct was satisfactory. Discriminant validity refers to the extent to which the constructs differ from one another empirically. It also measures the degree of difference between the overlapping constructs [56]. In the study, discriminant validity was evaluated using the Fornell-Larcker criterion and the heterotrait-monotrait (HTMT) ratio of correlations. The first method compares the square root of the AVE with the correlation of latent constructs [56]. A latent construct should explain the variance of its own indicator better than the variance of other latent constructs. Therefore, the square root of each construct’s AVE should have a greater value than the correlations with other latent constructs [57,58], and correlations among the different constructs may not exceed the threshold of 0.80 [59].

Table 3 shows the correlations among all the constructs of the proposed model and, along the diagonal, the AVE square root. Considering the results, there is discriminant validity among the constructs measured.

Although the common approach to assessing discriminant validity is the Fornell-Larcker criterion [59], a more precise measure of discriminant validity has recently been proposed—that is to say, the HTMT ratio of correlations, which compares each construct’s AVE with its squared consistent construct correlations. As shown in Table 4, the HTMT criterion (values below 0.90) [60] indicates that discriminant validity has been met. In sum, the discriminant validity results are acceptable using both methods.

### 4.4. Inner Model

The inner model is the structural relationship among constructs [61]. It includes an assessment of the pathways between latent constructs using linear regression in which the regressors can be interpreted as standardized beta coefficients. The confidence intervals of the path coefficients (based on bootstrapping of 500 samples) allow generalization of the results and the computation of Student’s t-test for each hypothesis. In terms of the model fit, the only approximate model fit criterion is the standardized root mean square residual (SRMR). SRMR is the square root of the sum of the squared differences between the model implied and the empirical correlation matrix. A value of zero for SRMR would imply a perfect fit and, generally, an SRMR value of less than 0.05 shows an acceptable fit [62].

**Figure 2:**
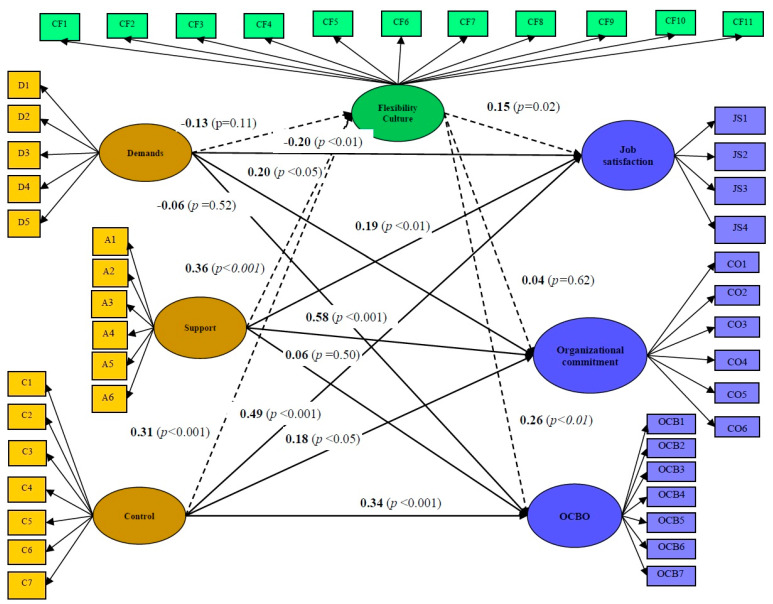
Structural model. Standardized Regression Coefficients (t values).

In the present study, the SRMR values for both the estimated model (0.104) and the saturated model (0.101) show an acceptable fit. SMART-PLS also provides additional fit indices [55], such as the Chi-Square (1.996) and the NFI (0.924). Although the results of the SRMR values, the chi-square test used in conjunction with NFI indices are sufficient to assess a model’s overall fit, similar results were found analyzing this factor structure using AMOS 5.0 (*RMSEA* = 0.084; *CFI* = 0.914; *NFI* = 0.922). In this model, we included the relationships among demands, support, and control on the one hand, and job satisfaction, organizational commitment, and OCBO on the other. As will be shown later in Figure 2, most of the direct effects of work demands, support, and job control on job satisfaction, organizational commitment and OCBO reached statistical significance (Hypotheses H1a, H1b, H2a, H2b, H3a, H3b, H3c are supported), except for two. There was not a significant effect of demands and support on OCBO. Consequently, H1c and H2c are not supported. As regards the indirect effects (Figure 2), results show that work demands had no significant effects on the flexibility-oriented culture (H4a is not supported). Nevertheless, results showed an indirect effect of support and control on job satisfaction and a flexibility-oriented culture on OCBO.

Beyond hypotheses testing, this part of the analysis also shows the variance explained (R^2^) in endogenous variables. This result indicates that all the exogenous variables—namely, demands, support and control—are expected to explain 50.5 percent of the variance in job satisfaction, 36.2 percent of the variance in organizational commitment, and 28.3 percent of the variance in OCBO. Furthermore, Table 5 shows the effect size (f^2^) value of each exogenous variable examined by following the instructions of Cohen [63] and indicators’ collinearity statistics. As can be seen, VIF values are lower than 5 and their tolerance values are higher than 0.2, so there is no collinearity problem [64,65].

In sum, the results of hypotheses testing are shown in Table 5 and Figure 2 below.

### 4.5. Suggesting Interventions: The Importance-Performance Matrix (IPMA)

We used an IPMA to complete the data analysis from PLS-PM. IPMA extends the standard results reporting of path coefficient estimates by adding a dimension that considers the average values of the latent variable scores. The aim is to identify predecessors that have high importance for the construct (i.e., those that have a strong total effect) but that also have low performance (i.e., low average latent variable scores) [62]. Expanding the analysis to the indicator level allows identification of the most important areas of specific actions, which would be particularly important to suggest interventions aimed at increasing employees’ job satisfaction, their OCBO, and their organizational commitment based on the relationship of these behaviors with perceptions of the job content (demands, control, and support) and flexibility-oriented culture.

**Figure 3:**
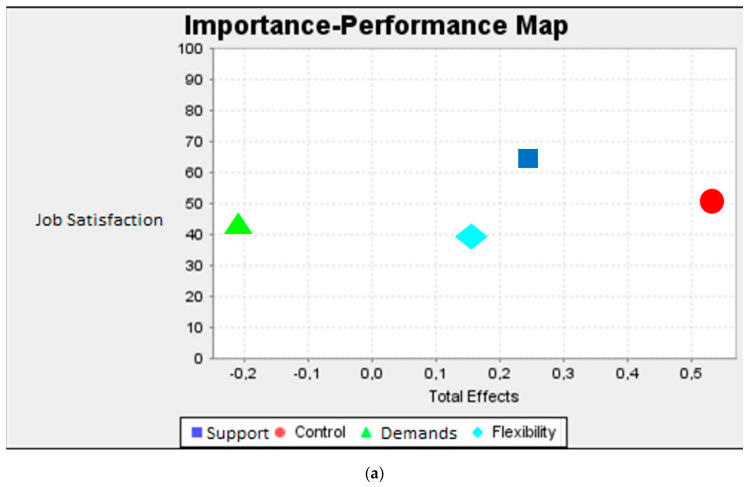

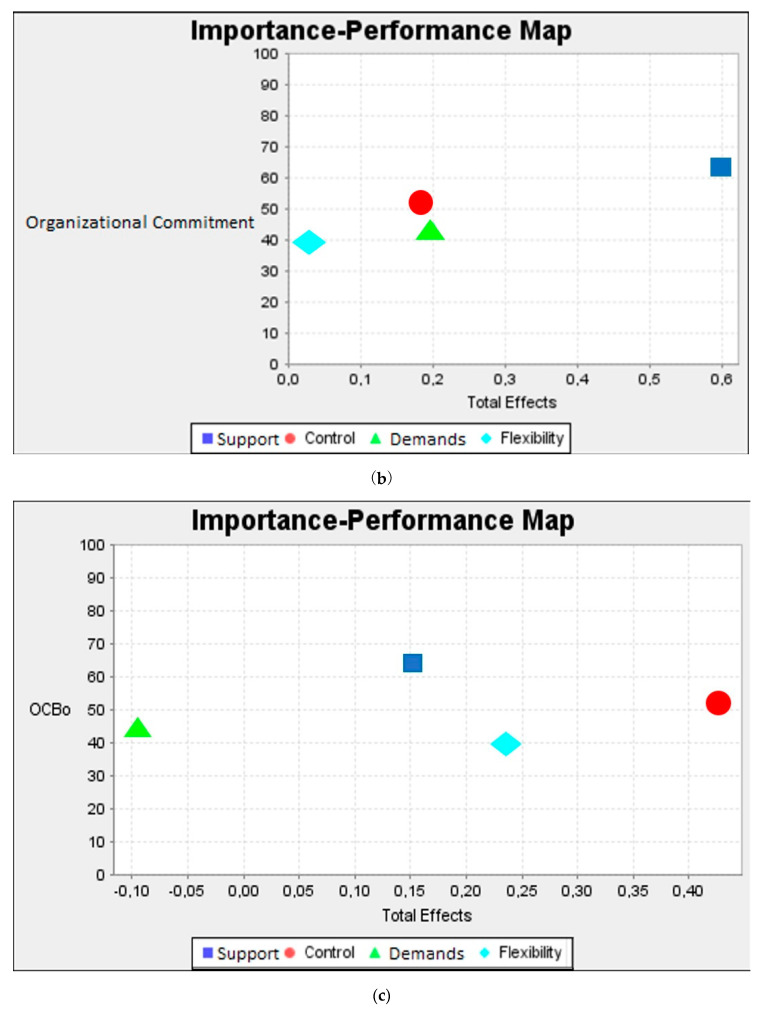
IPMA for support, control, demands, and flexibility-oriented culture on the three criterion variables. (**a**) IPMA on job satisfaction; (**b**) IPMA on organizational commitment; (**c**) IPMA on organizational citizenship behavior towards the organization.

In Figure 3a, the IPMA shows demands to be the most important predictor to understand job satisfaction. However, demands perform lower than average in comparison with the other constructs, especially control. When predicting organizational commitment (Figure 3b), a flexibility-oriented culture is relatively important, but support clearly performs relatively higher than the remaining constructs, although this dimension is less important to understand organizational commitment. When it comes to understanding OCBO (Figure 3c), support and a flexibility-oriented culture have a worse performance despite their importance, particularly when compared with control, which shows the opposite pattern.

In sum, these results could provide additional information on how to influence workers’ attitudes and behaviors based on their perceptions of job content and the flexibility-oriented organizational culture.

## 5. Discussion

This study aimed to analyze the influence of work demands and resources (support and control) on the attitudes and behaviors of Spanish police officers, considering the mediating role of a flexibility-oriented organizational culture. According to the results obtained, we can say that the proposed model supports almost all the hypothesized relationships between the constructs since most of them reach statistical significance (Table 6). Nevertheless, two—organizational commitment and flexibility-oriented organizational culture—are not significant.

Our findings match a vast line of research that supports the effects of work demands and resources on employee outcomes. More specifically, Bakker et al. [9,10] highlighted the role of resources as the only predictor of organizational commitment, which in turn acted as a mediator between work resources and the frequency of work absenteeism. Their study [8] goes beyond Karasek´s model, proposing that there can be multiple influences on the main variables, which would mean something more than a linear control of human resources, providing a complex net in the workplace, as is shown in the present work. In the same vein, Finnegan et al. [21] found that by increasing control over their work, employees tend to express higher levels of job satisfaction, more organizational commitment, and less intention of leaving their work, as well as feeling more psychologically empowered.

Regarding Karasek and Theorell´s model [18], Maes and Van der Doef [16] consider that the most important component of work demands is the workload. Although our results are not following this, it should be pointed out that in the case of police officers, work stressors and demands could be more related to interactions with citizens, victims or offenders, and other groups than to labor load. Therefore, it may be interesting to learn the value of the psychological workload for police officers instead of just countable work such as the number of complaints dispatched to Court in a week, for example.

Concerning our hypothesized model, a flexibility-oriented organizational culture plays a mediating role in the relationship between predictors and outcomes, such as job satisfaction and OCB of police officers. A flexibility-oriented organizational culture seems to be related to the attitudes and behaviors of police officers. What is much more important, though, is that these relationships have not previously been clearly established.

Therefore, the most relevant contribution of this paper is its focus on responding to the idea proposed by Bakker and Demerouti [9] concerning the need to consider the multi-level nature of data and to analyze perceptions shared by workers in units, departments, and companies. In this sense, a study such as this that attempts to integrate constructs at different levels can provide suggestions for designing more effective interventions [66]. In our opinion, these results are highly relevant not only because the model shows a good fit, but also because of the subsequent exploration employing the IPMA which allows us to draw conclusions about the influence of some predictors on others when predicting the attitudes and behaviors of police officers. Consequently, control and support (resources), when predicting employees’ outcomes, have a relatively high performance compared to the other predictors such as resources and a flexibility-oriented organizational culture, which provides opportunities for intervention. In this vein, some researchers agree on the importance of resources to obtain desirable outcomes for organizations, regardless of work demands [21]. For example, Karasek [18] suggests that work stress could be reduced by increasing control at work, regardless of work demands. Following the same reasoning, this author states that the adverse effects of low work control on job satisfaction are not limited to workers suffering high work demands but extend to workers doing passive jobs—that is to say, with low work demands and low control at work.

Hence the importance of resources to obtain organizational outcomes regardless of work demands is highlighted. As has been mentioned, work demands and a flexibility-oriented organizational culture are relatively important for job satisfaction and organizational commitment but perform lower than work resources (control and support), which proposes a way forward to improve professional development.

Nowadays, police officers should cope with cultural diversity in the communities that they develop their operations. Hence, police departments should attempt to provide their services with equal treatment and level of quality to every group of the community. Taking our empirical results into consideration, the importance of a flexibility-oriented organizational culture should be underlined as critical for the police to develop appropriate strategies to address community conflicts effectively. Especially, since the scope for action is sometimes too narrow and at other times is too wide has been positively valued by legal and policing agents because it provides a guideline. Nevertheless, in the absence of guidelines, share cultural knowledge can provide help, because it is the resource always at hand and trusted by officers, especially when a new regulation emerges.

Finally, the study’s limitations include its cross-sectional design, which restricts causal inferences, and the collection of self-reported data, which could generate methodological problems such as selection and information bias [67]. Additionally, some researchers consider that organizational culture would be more adequately conceptualized as a group- or organizational-level variable [68]. In this vein, their findings support that organizational culture could affect the strength of the relationship between independent variables and dependent variables. In this paper, we have focused on the individual perceptions of the flexibility orientation of culture, instead of calculating any aggregation index. Following the procedure of Azanza and colleagues [15], we have conducted the analyses considering that all the variables were set at a personal level. In the same vein, researchers suggested that organizational culture should be considered as a moderator in the JD-R model, instead of as a mediator [68]. As the evidence is not conclusive, in this study we have conceptualized the flexibility-oriented culture as a personal resource and, hence, could exert a mediator role according to Taris and Schaufeli [69]. Despite these limitations, this study can contribute to improve the wellbeing of people in organizations, as well as to achieve the performance goals of the Spanish police force.

## 6. Conclusions

Our study addressed the impact of job demands and resources (support and control) on relevant personal and organizational outcomes. On the one hand, as recent reviews stated, occupational stress can be severely damaging for employees [70] and individual resources, as psychological capital [71] or skills use [72], could help them in coping with job stress and its related outcomes. On the other, effective management of the negative emotions [73] during confronting stressful situations at work would be also a source of calm and peace for workers, but at the same time, it could be a hard duty for them [74]. Hence, promoting flexibility – oriented culture provides broad support for employees and allow us to expand the JD-R model including variables from organizational levels as other researchers recommended [68].

## Figures and Tables

**Table 1:** Sociodemographic data of the sample regarding gender.

	Men *N* = 107	Women *N* = 75	Total *N* = 182
Age			
Rank	24 to 61	29 to 59	24 to 61
*M* (*SD*)	43.59 (9.15)	44.17 (8.13)	43.83 (8.72)
Studies			
High school education	(*n* = 37) 34.57%	(*n =* 20) 26.7%	(*n =* 57) 31.31%
Professional training	(*n* = 31) 28.97%	(*n* = 18) 24%	(*n =* 49) 27%
University studies	(*n* = 39) 36.46%	(*n* = 37) 49.30%	(*n =* 76) 41.69%
Region (Comunidad Autónoma)			
Madrid	(*n* = 48) 44.9%	(*n* = 36) 48%	(*n* = 84) 46.15%
Castilla-León	(*n* = 31) 29%	(*n* = 22) 29.4%	(*n* = 53) 29.13%
Castilla La Mancha	(*n* = 28) 26.1%	(*n* = 17) 22.6%	(*n* = 45) 24.72%
Rank			
Upper Management	(*n* = 6) 5.61%	(*n* = 3) 4%	(*n* = 9) 5%
Executive Management	(*n* = 35) 32.71%	(*n* = 20) 26.67%	(*n* = 55) 30.22%
Police Officers	(*n* = 66) 61.68%	(*n* = 52) 69.33%	(*n* = 118) 64.78%
Years of service			
Rank	2 to 35 years	1 to 35 years	1 to 35 years
*M* (*SD*)	18.58 (9.34)	14.71 (9.78)	16.9 (*SD* = 9.7)

**Table 2:** Means, standard deviation, correlations (*N* = 182), and discriminant validity.

Variables	M	SD	1	2	3	4	5	6	7	8	9
1. Age	43.8	8.7	*-*								
2. Seniority	16.9	9.7	0.71 **	*-*							
3. Work Demands	2.8	0.75	−0.09	−0.00	*0.73*						
4. Social Support	3.6	0.72	−0.00	−0.22 **	−0.38 **	*0.78*					
5. Control	3.1	0.79	−0.14	0.04	0.11	0.16 *	*0.72*				
6. Flexibility-Oriented Culture	2.5	0.58	−0.06	−0.07	−0.24 **	0.48 **	0.35 **	*0.70*			
7. Job Satisfaction	3.1	0.79	−0.11	−0.00	−0.25	0.43 **	0.55 **	0.46 **	*0.84*		
8. Organizational Commitment	3.5	0.64	−0.15	−0.06	0.02	0.45 **	0.28 **	0.27 **	0.42 **	*0.72*	
9. OCBO	3.3	0.67	−0.12	0.06	−0.11	0.29 **	0.41 **	0.42 **	0.53 **	0.35 **	*0.71*

Note: OCBO: Organizational Citizenship Behavior Organization-oriented, Diagonal values are the squared root of AVE between the constructs and their indicators. For discriminant validity, diagonal values should be greater than off-diagonal values in the same row and column. * *p* < 0.05, ** *p* < 0.01.

**Table 3:** Outer model: Reliability and convergent validity results.

Latent Variables	Item	λ	*ρc*	α	AVE
Demands	D1.	0.72	0.85	0.81	0.54
	D2.	0.51			
	D3.	0.84			
	D4.	0.70			
	D5.	0.84			
Social Support	A1.	0.76	0.90	0.87	0.60
	A2.	0.77			
	A3.	0.76			
	A4.	0.83			
	A5.	0.72			
	A6.	0.82			
Control	C1.	0.62	0.87	0.85	0.52
	C2.	0.78			
	C3.	0.82			
	C4.	0.59			
	C5.	0.80			
	C6.	0.75			
	C7.	0.68			
Flexibility-Oriented Culture	CF1.	0.60	0.87	0.83	0.50
	CF2.	0.60			
	CF3.	0.53			
	CF4.	0.72			
	CF5.	0.73			
	CF6.	0.73			
	CF7.	0.54			
	CF8.	0.60			
	CF9.	0.60			
	CF10.	0.62			
	CF11.	0.74			
Job Satisfaction	SL1.	0.87	0.90	0.86	0.70
	SL2.	0.74			
	SL3.	0.90			
	SL4.	0.82			
Organizational Commitment	CO1.	0.78	0.86	0.81	0.52
	CO2.	0.68			
	CO3.	0.67			
	CO4.	0.80			
	CO5.	0.61			
	CO6.	0.75			
OCBO	OCB1.	0.66	0.88	0.84	0.51
	OCB2.	0.60			
	OCB3.	0.75			
	OCB4.	0.67			
	OCB5.	0.78			
	OCB6.	0.70			
	OCB7.	0.79			

Note: OCBO: Organizational Citizenship Behavior Organization-oriented.

**Table 4:** Outer model: Discriminant validity results following HTMT.

	*1*	*2*	*3*	*4*	*5*	*6*
1. Demands						
2. Support	0.43					
3. Control	0.29	0.27				
4. Flexibility-Oriented culture	0.33	0.61	0.39			
5. Job Satisfaction	0.31	0.48	0.64	0.56		
6. Organizational Commitment	0.19	0.61	0.35	0.38	0.51	
7. OCBO	0.22	0.33	0.50	0.48	0.65	0.42

Note: OCBO: Organizational Citizenship Behavior Organization-oriented.

**Table 5:** Collinearity Statistics and Effect Size (f^2^).

	Collinearity Statistics		Effect Size (f^2^)-f-Squared f^2^		
			Job Satisfaction	Organizational Commitment	OCBO
	Tolerance	VIF	R-Squared	R-Squared	R-Squared
			0.505 (50.5%)	0.362 (36.2%)	0.283 (28.3%)
Demands	0.829	1.207	0.041	0.004	0.008
Support	0.818	1.223	0.163	0.246	0.091
Control	0.942	1.062	0.301	0.112	0.184

Note: f^2^ of 0.02, 0.15, and 0.35 can be viewed as a gauge for whether a predictor has a small, medium, or large effect at the structural level.

**Table 6:** Results of hypotheses testing (*N* = 182).

Model with Direct Effects				
*H1a*	Work demands		Job Satisfaction	**Supported**
*H1b*	Work demands		Organizational Commitment	**Supported**
*H1c*	Work demands		OCBO	Not supported
*H2a*	Perceived Support		Job Satisfaction	**Supported**
*H2b*	Perceived Support		Organizational Commitment	**Supported**
*H2c*	Perceived Support		OCBO	Not supported
*H3a*	Job Control		Job Satisfaction	**Supported**
*H3b*	Job Control		Organizational Commitment	**Supported**
*H3c*	Job Control		OCBO	**Supported**
**Model with Indirect Effects**				
*H4a*	Work demands	Flexibility-oriented culture	Job Satisfaction	Not supported
			Organizational Commitment	Not supported
			OCBO	Not supported
*H4b*	Perceived Support	Flexibility-oriented culture	Job Satisfaction	**Supported**
			Organizational Commitment	Not supported
			OCBO	**Supported**
*H4c*	Job Control	Flexibility-oriented culture	Job Satisfaction	**Supported**
			Organizational Commitment	Not supported
			OCBO	**Supported**

Note: OCBO: Organizational Citizenship Behavior Organization-oriented.
